# The impact of baseline body mass index on clinical outcomes in metastatic breast cancer: a prospective study

**DOI:** 10.1186/s13104-017-2876-2

**Published:** 2017-11-02

**Authors:** Hiba Alarfi, Maher Salamoon, Mohammad Kadri, Moosheer Alammar, Mhd Adel Haykal, Alhadi Alseoudi, Lama A. Youssef

**Affiliations:** 1 Program of Clinical and Hospital Pharmacy, School of Pharmacy, University of Damascus, Damascus, Syria; 2 AlBaironi Hospital, Ministry of Higher Education, Damascus, Syria; 3 Biomedical Sciences Program, School of Pharmacy, University of Damascus, Damascus, Syria

**Keywords:** Metastatic breast cancer, Body mass index, Response, Survival

## Abstract

**Objective:**

The prognostic value of body mass index (BMI) in metastatic breast cancer (MBC) has not been fully elucidated. In a prospective study to investigate the chemo-sensitizing effect of statins on clinical outcomes in MBC patients who were scheduled to receive palliative chemotherapy (Carboplatin and Vinorelbine), we sought to investigate the relationship between baseline BMI and clinical outcomes; response, overall survival (OS) and progression free survival (PFS), over a median follow-up of 40-months.

**Results:**

Eighty-Two MBC patients were enrolled and categorized using baseline BMI as underweight (BMI, < 18.5 kg/m^2^, n = 1), normal-weight (BMI, 18.5–24.9 kg/m^2^, n = 20), overweight (BMI, 25–29.9 kg/m^2^, n = 34), and obese (BMI, ≥ 30 kg/m^2^, n = 27). Median OS was 10 months in normal/underweight, 19 months in overweight, and 16 months in obese (*P* = 0.083). Univariate Cox model revealed that overweight patients were significantly less likely to die of MBC as normal BMI patients (hazard ratio [HR] = 0.54, 95% confidence interval [CI], (0.29–0.98), *P* = 0.044). Similarly, multivariate Cox model, after adjusting for age, number of metastatic sites, chemotherapy line’s grade, HER2 and hormone receptors status, confirmed longer survivorship of overweight in comparison with normal BMI patients (HR = 0.51, 95% CI (0.26–0.99), *P* = 0.047). Our data suggest that being overweight could improve OS in MBC patients.

**Electronic supplementary material:**

The online version of this article (10.1186/s13104-017-2876-2) contains supplementary material, which is available to authorized users.

## Introduction

Metastatic breast cancer (MBC) is a treatable but still generally incurable disease. The current treatment outcomes for MBC encompass relieving cancer-related symptoms, improving or maintaining quality of life, and possibly prolonging survival [1]. Median survival time for patients with MBC varies greatly due to the heterogeneity of MBC patients whose clinical outcome and prognosis may depend on variety of predictive factors [2, 3]. Obesity is a well-known risk factor for the development of postmenopausal breast cancer [4]. Numerous studies have demonstrated adiposity to be associated with recurrence and poorer survival among pre- and post-menopausal breast cancer patients [5–7]. The hypothesized mechanism by which overweight and obesity may affect prognosis in early-stage tumors involves stimulating breast cancer growth and progression via a multitude of factors including insulin resistance, increased estrogen synthesis and alteration of production of adipokines and cytokines [8]. Based on the association between high BMI and bad prognosis in women with early breast cancer, a similar association is speculated to exist in patients with MBC. Nevertheless, such an association between BMI and prognosis in MBC patients is debatable and only scarce direct evidence supporting or refuting such an effect is available [2, 9, 10]. Given the lack of consensus in the literature, we aimed at exploring the impact of BMI on survival and response to treatment in MBC patients on palliative chemotherapy.

Investigating the impact of BMI on clinical outcomes was one secondary goal of our study to assess the chemo-sensitizing effects of Statin in MBC patients.

## Main text

### Patients and methods

The study protocol was approved by the Scientific Research Ethics Committee at the Faculty of Pharmacy, Damascus University and the Scientific Board of Al-Baironi Hospital. Eligibility criteria included confirmed diagnosis of MBC (stage IV) prior to commencing chemotherapy course consisting of Carboplatin and Vinorelbine, age older than 18 years, and Eastern Cooperative Oncology Group Performance Status (ECOG-PS) ≤ 2. All eligible patients gave signed informed consent. Enrollment started in August 2011 and ended in July 2012, and the follow-up lasted until death or the cutoff date of December 2015. Body Mass Index (kg/m^2^) was assessed at baseline, and patients were categorized according to the World Health Organization definition: underweight < 18.5 kg/m^2^, normal-weight 18.5–24.9 kg/m^2^, overweight 25–29.9 kg/m^2^ and obese ≥ 30 kg/m^2^. Due to the low number of underweight patients (n = 1), we merged the underweight group with the normal-weight group under normal BMI (< 25 kg/m^2^) for the purpose of simplifying the comparisons.

### Treatment line and response assessment

Chemotherapy regimen was conducted every 3 weeks according to the hospital protocol as follow; Carboplatin (Carboplatin “Ebewe”) IV (Area under the curve: AUC 4) on day 1 and Vinorelbine (Navelbine^®^) IV (25 mg/m^2^) or orally (60 mg/m^2^) on days 1 and 8 in each cycle, for a median of 6 cycles. Additionally, patients with bone metastases were treated with zoledronic acid via intravenous infusion (4 mg over at least 15 min every 4 weeks). Treatment related-Toxicity was graded according to the Common Terminology Criteria for Adverse Events, version 4. Treatment response assessment was performed every 3 cycles. Patients’ response was classified according to the Response Evaluation Criteria in Solid Tumor (RECIST) (version 1.1) as follow; complete response (CR), complete disappearance of clinical evidence of disease for a minimum of 8 weeks; partial response (PR), decreased in tumor burden ≥ 30%; stable disease (SD), decreased by < 30% or increased by < 20%; progressive disease (PD), increased in tumor burden by ≥ 20%. Due to the paucity of patients who demonstrated complete response to therapy, we combined CR and PR in one group under objective response. Overall survival (OS) was defined as time from study entry to death from any cause. Progression free survival (PFS) was defined as the time interval from study entry to disease progression, or death from any cause, whichever occurred first.

### Statistical analysis

Statistical analysis was performed using Graphpad Prism^®^ (version 5) except for proportional hazard Cox regression models that were performed using SPSS^®^ (version 22) to calculate hazard ratios (HRs) and 95% confidence intervals (CIs) for both univariate and multivariate analyses. We assessed the associations between BMI categories and patients’ characteristics using Chi square test for categorical variables and One-way ANOVA for continuous variables. Median overall survival and progression free survival were estimated using Kaplan–Meier method. Statistical significance was tested using the Log-rank test, and two-tailed *P* < 0.05 was considered significant. The median follow-up was estimated using reverse censoring for overall survival.

### Results

#### Descriptive information

Eighty-two eligible metastatic breast cancer patients were enrolled in this study. At the time of enrollment, one patient (1.22%) was underweight, 20 patients (24.39%) were normal-weight, 34 patients (41.46%) were overweight, and 27 patients (32.93%) were obese. Median age was 47.5 years (range from 24 to 74 years). Fifty-six patients (68.29%) were HER2 positive (Human epidermal growth factor receptor-2), and 39 patients (47.56%) were ER/PR negative (Estrogen receptor/Progesterone receptor). Forty patients (48.78%) had one metastatic site, whereas 42 patients (51.22%) had ≥ 2 metastatic sites. The chemotherapy regimen was the first line in 37 patients (45.12%) and the second in 36 (43.9%). Obese and overweight patients had a higher median age at diagnosis (*P* = 0.014). No significant differences were found between BMI categories with regard to baseline HER2 and hormone receptor status, number and sites of metastases, chemotherapy lines’ grade, or ECOG-PS. Patients’ demographic characteristics are summarized in Table 1.

**Table 1:** Baseline patients’ characteristics by BMI category

Characteristic	Total (n = 82)	BMI (kg/m^2^)			*P* value
		<25 (n = 21)	25–29.9 (n = 34)	≥ 30 (n = 27)	
*Age (years)*					
Median (range)	47.5 (24–74)	41 (24–71)	46 (28–74)	50 (36–68)	0.014
*ECOG-PS scale*	n%				
0	8 (9.76)	2 (9.52)	2 (5.88)	4 (14.81)	0.108
1	60 (73.17)	13 (61.90)	30 (88.23)	17 (62.96)	
2	14 (17.07)	6 (28.57)	2 (5.88)	6 (22.22)	
*No. of metastatic*					
1 site	40 (48.78)	8 (38.09)	15 (44.11)	17 (62.96)	0.180
≥ 2 sites	42 (51.22)	13 (61.90)	19 (55.88)	10 (37.04)	
*HER2*					
HER2+	56 (68.29)	10 (47.62)	25 (73.52)	21 (77.77)	0.064
HER2−	22 (26.82)	10 (47.62)	6 (17.64)	6 (22.22)	
Unknown	4 (4.88)	1 (4.76)	3 (8.82)	0 (0)	
*Hormone receptors*					
ER+PR+	27 (32.92)	8 (38.09)	13 (38.23)	6 (22.22)	0.613
ER−PR−	39 (47.56)	8 (38.09)	14 (41.17)	17 (62.96)	
ER+PR−	6 (7.31)	2 (9.52)	2 (5.88)	2 (7.41)	
ER−PR+	6 (7.31)	2 (9.52)	2 (5.88)	2 (7.41)	
Unknown	4 (4.88)	1 (4.76)	3 (8.82)	0 (0)	
*Chemotherapy lines*					
1st line	37 (45.12)	11 (52.38)	15 (44.11)	11 (40.74)	0.836
2nd line	36 (43.90)	7 (33.33)	16 (47.05)	13 (48.14)	
≥ 3rd line	9 (10.98)	3 (14.28)	3 (8.82)	3 (11.11)	
*Other medications*					
Statins	41 (50)	10 (47.62)	15 (44.11)	16 (59.26)	
*Metastatic sites*	(n = 129) sites	(n = 35)	(n = 55)	(n = 39)	
Bone	39 (30.23)	12 (34.29)	16 (29.09)	11 (28.21)	0.169
Visceral	61 (47.29)	18 (51.43)	29 (52.73)	14 (35.90)	
Soft tissues	29 (22.48)	5 (14.29)	10 (18.18)	14 (35.90)	

#### BMI and patients’ outcomes

Of the 82 patients enrolled in our study, only 77 patients (93.9%) were assessable by the end of the chemotherapy course; as 2 patients (2.4%) withdrew consent and 3 patients (3.7%) were classified inevaluable for response due to early death (n = 2) or chemotherapy toxicity (n = 1). Thirty-eight patients with bone metastasis received zoledronic acid in addition to the chemotherapy line. Overweight patients constituted the majority of responsive patients (55.56%), whereas most progressive patients were obese (44.83%) (see Additional file 1: Table S1).

Obese patients were less likely to experience chemotherapy-related toxicity (grade 3 or 4) with 21 out of 27 (77.78%) obese patients did not experience any type toxicity (grade 3 or 4) in comparison with 18/32 (56.25%) overweight patients, and 9/21 (42.86%) normal BMI patients.

**Fig. 1:**
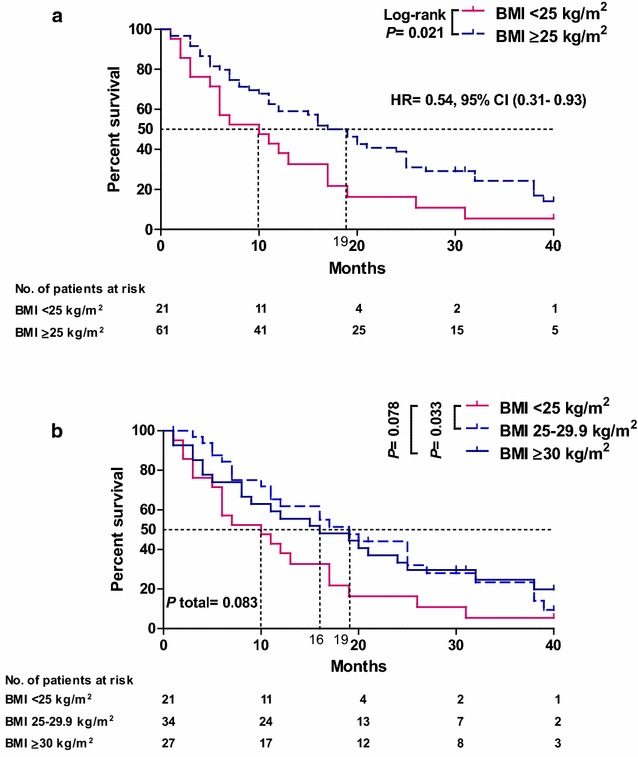
Survival curves with reference to baseline BMI for MBC patients who were treated with palliative chemotherapy (Carboplatin and Vinorelbine). Median survival was estimated during a 40-month follow up period using Kaplan–Meier method. Statistical significance was assessed using the Log-rank test and two-tailed *P* value of < 0.05 was considered significant. **a** Patients were categorized into two groups; normal BMI < 25 kg/m^2^ and high BMI ≥ 25 kg/m^2^. **b** High BMI patients were dissected into overweight (25–29.9 kg/m^2^) and obese (≥ 30 kg/m^2^), and survival of both subgroups was assessed in comparison with normal BMI

By the end of a median follow-up period of 40 months (range, 10–49 months), 65 fatalities were recorded, and the survival rates were 5.44, 9.34, 19.75% in normal BMI, overweight and obese patients, respectively. High BMI (≥ 25 kg/m^2^) patients had significantly better survival (19 months) when compared with normal BMI patients (BMI < 25 kg/m^2^) (10 months) (*P* = 0.021) (Fig. 1a). When high BMI category was dissected into obese and overweight groups, only overweight patients demonstrated statistically significant longer median survival (19 months) than normal BMI patients (*P* = 0.033), whereas the relatively better survival outcomes (16 months) in obese patients did not reach statistical significance (*P* = 0.078) (Fig. 1b). Univariate Cox models of survival among the three BMI categories revealed that overweight patients were approximately one half less likely to die of MBC than patients who had normal BMI (HR = 0.54, 95% CI (0.29–0.98), *P* = 0.044). A similar trend was observed for obese patients who showed longer survival than did normal BMI patients (HR = 0.55, 95% CI (0.29–1.02), *P* = 0.059). After adjusting for other factors, multivariate Cox models proved that overweight patients were still significantly less likely to die of MBC than normal BMI patients (HR = 0.51, 95% CI (0.26–0.99), *P* = 0.047) Nevertheless, no significant difference was observed between obese and normal BMI patients (HR = 0.54, 95% CI (0.26–1.15), *P* = 0.109). Furthermore, we assessed the prognostic significance of BMI within subgroups defined by age, number of metastatic sites, chemotherapy line’s grade, HER2 and hormone receptors status. We found that overweight patients were still significantly less likely to die of MBC than normal BMI patients only within the group of chemotherapy being ≥ 2nd line (HR = 0.38, 95% CI (0.15–0.92), *P* = 0.032) as it shown in Table 2.

**Table 2:** Results of proportional hazard Cox regression models for overall survival among overweight and obese patients in comparison with normal BMI patients

Variable	Univariate			Multivariate		
	HR^a^	(95% CI)	*P* value	HR^a^	(95% CI)	*P* value
*All patients*						
Overweight	0.54	(0.29–0.98)	*0.044*	0.51	(0.26–0.99)	*0.047*
Obese	0.55	(0.29–1.02)	0.059	0.54	(0.26–1.15)	0.109
Age (years) ≥ 50						
Overweight	0.31	(0.06–1.56)	0.155	0.28	(0.05–1.52)	0.140
Obese	0.37	(0.08–1.81)	0.221	0.26	(0.05–1.45)	0.125
*Age (years) < 50*						
Overweight	0.69	(0.35–1.35)	0.279	0.57	(0.28–1.17)	0.128
Obese	0.79	(0.35–1.78)	0.569	0.60	(0.22–1.60)	0.309
*≥2 metastatic sites*						
Overweight	0.73	(0.34–1.58)	0.432	0.67	(0.28–1.56)	0.349
Obese	1.28	(0.54–3.07)	0.574	1.43	(0.54–3.83)	0.473
*1 metastatic site*						
Overweight	0.37	(0.14–1.01)	0.051	0.49	(0.13–1.80)	0.279
Obese	0.36	(0.14–0.92)	*0.033*	0.31	(0.07–1.25)	0.099
*≥ 2nd line*						
Overweight	0.47	(0.21–1.05)	0.065	0.37	(0.15–0.92)	*0.032*
Obese	0.49	(0.21–1.15)	0.100	0.48	(0.17–1.34)	0.163
*1st line*						
Overweight	0.53	(0.21–1.35)	0.183	0.97	(0.36–2.64)	0.953
Obese	0.48	(0.18–1.29)	0.146	1.60	(0.35–7.39)	0.549
*Hormone receptors (−)*						
Overweight	0.68	(0.25–1.85)	0.450	0.71	(0.23–2.17)	0.548
Obese	0.57	(0.21–1.54)	0.269	1.07	(0.34–3.39)	0.910
*Hormone receptors (+)*						
Overweight	0.60	(0.27–1.33)	0.211	0.46	(0.16–1.28)	0.135
Obese	0.64	(0.26–1.57)	0.335	0.37	(0.10–1.30)	0.120
*HER2 (−)*						
Overweight	0.36	(0.10–1.24)	0.104	0.42	(0.11–1.68)	0.221
Obese	0.52	(0.16–1.69)	0.279	0.58	(0.06–5.14)	0.622
*HER2 (+)*						
Overweight	0.66	(0.30–1.46)	0.307	0.56	(0.24–1.32)	0.188
Obese	0.51	(0.22–1.18)	0.116	0.57	(0.23–1.44)	0.235

^a^Hazard ratios (HR) represent risk of death compared with normal BMI (< 25 kg/m^2^) patients

Median PFS was comparable among the three BMI groups; normal BMI (4 months), overweight (5.5 months), and obese patients (4 months) (P = 0.340). The hazard ratios (HR) for progression were 0.68 (95% CI (0.39–1.18), *P* = 0.173) for overweight and 0.91 (95% CI (0.51–1.62), *P* = 0.735) for obese, compared with normal BMI patients.

### Discussion

Metastatic breast cancer (MBC) is a heterogeneous disease with unpredictable clinical behavior [1]. Adiposity is an established risk factor for recurrence and poor survival in early-stage breast cancer [5, 7], however the link between obesity and treatment outcomes in patients with MBC remains unclear. Our work is one of the few to examine the plausible associations between BMI and clinical outcomes in MBC patients during treatment with palliative chemotherapy.

In the current study, we observed a trend towards an association between high BMI categories and better prognosis in patients with MBC receiving systemic chemotherapy. Contrary to the current dogma in early-stage breast cancer, our data suggest that normal BMI in metastatic disease is associated with increased risks of death compared with overweight or obese. Moreover, chemotherapy related toxicity was more prevalent in normal BMI patients when compared with overweight and obese patients. Our findings also demonstrate a trend towards a higher objective response rates and lower HR of disease progression among overweight and obese patients compared with normal BMI patients. The few studies that assessed the prognostic value of BMI in advanced breast cancer have yielded conflicting results. In line with our findings, Gennari et al. showed a trend toward improved PFS and OS in overweight patients when compared with normal weight and obese patients, and therefore suggested that being overweight should not be regarded as an adverse prognostic factor in patients with MBC [10]. To the contrary, von Drygalski et al. reported an association between obesity and decreased survival in MBC patients who underwent stem cell support following high-dose chemotherapy [9]. In a study by Jung et al. better prognosis was reported to be limited to underweight women (BMI < 20 kg/m^2^), while no difference seen between normal weight, overweight, and obese patients [2].

Our findings are in conflict with data from previous studies on early-stage breast cancer patients in which a strong association between increased BMI and worse prognosis was observed [11, 12]. Moreover, obesity was reported to negatively impact prognosis when patients of all stages of breast cancer including MBC were combined [13, 14]. We cannot fully explain this discrepancy, but clues may arise from the recently emerged “obesity paradox”. According to this paradigm, obesity plays a crucial role in the development of many acute and chronic diseases, however higher BMI may have protective advantage, and even survival benefits, in individuals with advanced cancers and other acute or chronic diseases [15].

If all-cause mortality is to be considered, obesity seems to confer a survival advantage rather than a disadvantage in patients with diseases associated with wasting, including cancer [16, 17]. Moreover, ongoing loss of weight and severe muscle depletion were independent indicators of poor prognosis, even for obese individuals.

Further support to our results originate from several studies on patients with solid tumors other than breast cancer. In two large phase III studies (CAIRO and CAIRO2), which involved advanced colorectal cancer (ACC) patients, Simkens et al. described an association between higher BMI and better median OS [18]. Additionally, Montgomery et al. observed that higher BMI at baseline was a significant predictor of better response and survival for patients with advanced androgen dependent prostate cancer [19]. Furthermore, Hakimi et al. reported that overweight and obese patients with clear-cell renal cell carcinoma (ccRCC) were more likely to present with less-aggressive tumors and reduced risk of RCC death when compared with normal weight patients [20]. Intriguingly, these counterintuitive findings are seemingly consistent with the obesity paradox, since the risk of primary colorectal cancer, renal cell carcinoma and relapse and/or mortality from prostate cancer increases with higher BMI [4].

In conclusion, our findings imply a protective role of high BMI in metastatic breast cancer. This study provides greater impetus for further investigation of the relationship between BMI and treatment outcomes in MBC.

## Limitations

Our study’s main limitation is the small sized sample of MBC patients receiving one line of chemotherapy (Carboplatin and Vinerolbine).

## Additional file

**Additional file 1: Table S1.** The treatment efficacy’s outcomes in each BMI group. Description of data: overall response outcomes and PFS according to BMI categories.

## Abbreviations

BMI: body mass index
MBC: metastatic breast cancer
OS: overall survival
PFS: progression free survival
HR: hazard ratio
ECOG-PS: eastern cooperative oncology group performance status
AUC: area under the curve
RECIST: response evaluation criteria in solid tumor
CR: complete response
PR: partial response
SD: stable disease
PD: progressive disease
ER: estrogen receptor
PR: progesterone receptor
HER2: human epidermal growth factor receptor-2
ACC: advanced colorectal cancer
ccRCC: clear-cell renal cell carcinoma
