# Supplementary material for: The impact of baseline body mass index on clinical outcomes in metastatic breast cancer: a prospective study
Source: BMC Res Notes. 2017 Nov 2;10:550. doi: 10.1186/s13104-017-2876-2 (PMC5667491; doi:10.1186/s13104-017-2876-2)
Supplement: Supplementary file 1 — Additional file 1: Table S1. The treatment efficacy’s outcomes in each BMI group. Description of data: overall response outcomes and PFS according to BMI categories. [file 13104_2017_2876_MOESM1_ESM.docx]

| **Table S1.** **The treatment efficacy's outcomes in each BMI group** | | | | |
| --- | --- | --- | --- | --- |
| **Outcomes** | **BMI (kg/m^2^)** | | | ***P* value** |
|  | **<25** | **25-29.9** | **≥30** |  |
| **No. of treatment cycles** |  |  |  | **0.103** |
| Median | 3 | 6 | 4 |  |
| (Minimum- Maximum) | 1-12 | | |  |
| **Progression Free Survival (PFS)** |  |  |  |  |
| Median PFS (months) | 4 | 5.5 | 4 | **0.340** |
| HR* (95% CI) | 1 (reference) | 0.68 (0.39-1.18) | 0.91 (0.51-1.62) |  |
| **Overall Response#** |  | n (%) |  | **0.275** |
| Objective response (total=27) | 4 (14.81) | 15 (55.56) | 8 (29.63) |  |
| Stable disease (total=21) | 7 (33.33) | 8 (38.09) | 6 (28.57) |  |
| Progressive disease (total=29) | 7 (24.14) | 9 (31.03) | 13 (44.83) |  |
| ^#^ The three patients who were classified inevaluable for response were normal BMI (<25 kg/m^2^), and the two withdrawn patients were overweight  * Hazard ratios (HR) are unadjusted for other factors | | | | |
